# Breathlessness in COPD: linking symptom clusters with brain activity

**DOI:** 10.1183/13993003.04099-2020

**Published:** 2021-11-18

**Authors:** Sarah L. Finnegan, Olivia K. Harrison, Catherine J. Harmer, Mari Herigstad, Najib M. Rahman, Andrea Reinecke, Kyle T.S. Pattinson

**Affiliations:** 1 Wellcome Centre for Integrative Neuroimaging and Nuffield Division of Anaesthetics, Nuffield Dept of Clinical Neurosciences, University of Oxford, Oxford, UK; 2 Translational Neuromodeling Unit, Institute for Biomedical Engineering, University of Zurich and ETH Zurich, Zurich, Switzerland; 3 School of Pharmacy, University of Otago, Dunedin, New Zealand; 4 Dept of Psychiatry, Medical Sciences Division, University of Oxford, Oxford, UK; 5 Oxford Health NHS Foundation Trust, Warneford Hospital, Oxford, UK; 6 Dept of Biosciences and Chemistry, Sheffield Hallam University, Sheffield, UK; 7 Nuffield Dept of Medicine, University of Oxford, Oxford, UK; 8 NIHR Oxford Biomedical Research Centre, Oxford, UK

## Abstract

**Background:**

Current models of breathlessness often fail to explain disparities between patients' experiences of breathlessness and objective measures of lung function. While a mechanistic understanding of this discordance has thus far remained elusive, factors such as mood, attention and expectation have all been implicated as important modulators of breathlessness. Therefore, we have developed a model to better understand the relationships between these factors using unsupervised machine learning techniques. Subsequently we examined how expectation-related brain activity differed between these symptom-defined clusters of participants.

**Methods:**

A cohort of 91 participants with mild-to-moderate chronic obstructive pulmonary disease (COPD) underwent functional brain imaging, self-report questionnaires and clinical measures of respiratory function. Unsupervised machine learning techniques of exploratory factor analysis and hierarchical cluster modelling were used to model brain–behaviour–breathlessness links.

**Results:**

We successfully stratified participants across four key factors corresponding to mood, symptom burden and two capability measures. Two key groups resulted from this stratification, corresponding to high and low symptom burden. Compared with the high symptom burden group, the low symptom burden group demonstrated significantly greater brain activity within the anterior insula, a key region thought to be involved in monitoring internal bodily sensations (interoception).

**Conclusions:**

This is the largest functional neuroimaging study of COPD to date, and is the first to provide a clear model linking brain, behaviour and breathlessness expectation. Furthermore, it was possible to stratify participants into groups, which then revealed differences in brain activity patterns. Together, these findings highlight the value of multimodal models of breathlessness in identifying behavioural phenotypes and for advancing understanding of differences in breathlessness burden.

## Abstract 2

**Towards individualised treatments for chronic breathlessness with functional neuroimaging: revealing the factors underlying the breathlessness experience in COPD** https://bit.ly/3a8fXPt

## Introduction

For the millions of people living with chronic obstructive pulmonary disease (COPD), asthma, heart failure, cancer and survivors of intensive care, chronic breathlessness is a major source of suffering. Breathlessness extends pervasively into people's lives, and carries substantial personal, social and economic impact [1–3]. Adding to the burden of COPD and impeding its effective clinical care is the subjective nature of breathlessness, which corresponds poorly with objective disease markers and clinical estimations of severity [4]. While traditional models of breathlessness fail to explain this discordance, recent work in asthma [5] has highlighted that the one-to-one coupling between symptom and disease severity exists only as a small part of a wider spectrum. At one end of this spectrum a person with relatively mild disease may find themselves disabled by feelings of breathlessness (symptom predominant), while conversely, others with severe disease may suffer less and have a considerably better quality of life (*e.g.* inflammation predominant) [5].

While conventional thinking has viewed breathlessness as a symptom arising directly from lung disease or cardiopulmonary stress, neuroimaging studies are beginning to reveal the key role of the brain in generating and maintaining breathlessness perception [6–9]. Within these models, breathlessness may not simply arise from the current patho-physiological status of the lungs and airways, but rather as a complex product of previous experiences, expectations, emotional state and perception of internal sensory signals [10].

The importance of considering breathlessness as a multisystem experience is stressed by the 2012 American Thoracic Society statement, which highlighted the link between the neural systems subserving breathlessness and that of chronic pain [3]. While the field of chronic pain research has taken strides towards generating neuro-biomarkers [11–14], which may sit within individualised treatment pathways in the future [15], similar applications in chronic breathlessness are in their infancy. The foundations for this work are in place; stratification techniques have been used in COPD, asthma and other cardiorespiratory diseases to examine patients in terms of comorbidities and clinically relevant groupings of physiology and psychology [5, 10, 16, 17]. Additionally, neuro-psychological phenotypes have been proposed for asthma [18]. However, data-driven models exploring the psychological factors of breathlessness and their variability across individuals have yet to be formalised in COPD.

Experimental studies in both healthy adults and patients with COPD have used both resistive loads [8, 19–25] and air hunger [26–28] to examine breathlessness-related brain activity. Of these methods, air hunger is thought to most closely mirror the unpleasantness experienced in chronic breathlessness [29]. However, as breathlessness is a multidimensional experience [3, 30, 31], the approach taken should differ depending on the dimension under scrutiny and the clinical translation in mind. Thus, while laboratory-induced sensational cues and breathing work/effort may examine the physical experience of breathlessness, this study focuses on other important aspects of breathlessness, including the long-term lived experience of breathlessness perception, expectation and emotional responses.

A body of evidence now links symptom perception with expectation [32–34]. This is particularly relevant for populations with chronic symptoms of breathlessness, where the emotion and expectation of breathlessness–cue relationships are deeply complex and entrenched. Even in healthy volunteers, the expectation alone of a breathless-inducing situation, such as an odorous gas, can be sufficient to drive brain activity patterns and resulting breathlessness in the absence of afferent input [34].

Here we translated an experimental anticipation paradigm, often undertaken in healthy volunteers, into a more clinically relevant design. In doing so we aimed to better understand breathlessness-expectation-related brain activity and the interaction between behavioural, physiological and psychological factors of breathlessness, within the individual. To achieve this, we applied unsupervised machine learning techniques to outcomes derived from a cohort of 91 patients with COPD, using standardised measurements of physiology as well as comprehensive psychological and behavioural tests. We used these measures to examine the relationship between brain activity, behaviour and breathlessness across individuals, and aimed to stratify participants into potentially therapeutically relevant groupings.

## Methods and materials

### Participants

**FIGURE 1:**
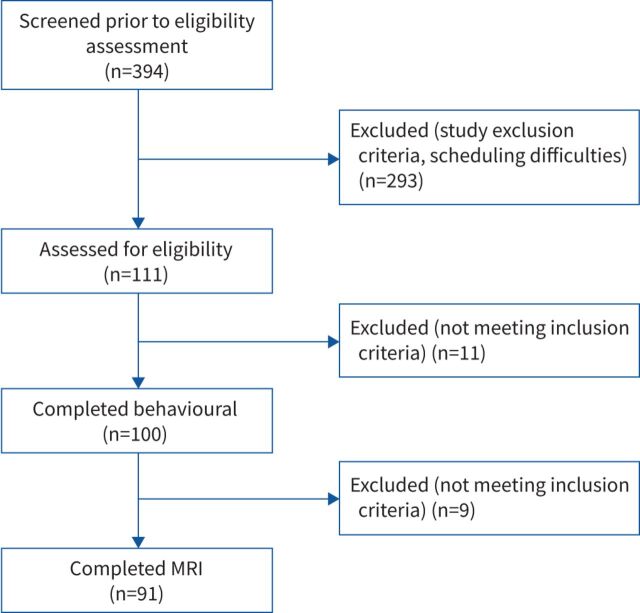
Flowchart of the stages of participant recruitment, reasons for exclusion and total number of completed participant datasets. MRI: magnetic resonance imaging.

100 participants (36 females, median (range) age 70 (49–84) years) with mild-to-moderate COPD (according to Global Initiative for Chronic Obstructive Lung Disease standards) were recruited immediately prior to their enrolment in a National Health Service-prescribed course of pulmonary rehabilitation. From this population, 91 participants completed the magnetic resonance imaging (MRI) component of the study (table 1). Reasons for non-completion of the MRI scan are detailed in figure 1. The data presented here correspond to the participants’ first (baseline) study visit as part of a wider investigation of the effects of d-cycloserine on pulmonary rehabilitation outcome (to be published separately). Written informed consent was obtained prior to the start of the study. Study approval was granted by South Central Oxford Research Ethics Committee B (118784). Study inclusion criteria included a diagnosis of COPD and admittance to pulmonary rehabilitation. Exclusion criteria included inadequate understanding of verbal and written English, significant cardiac, psychiatric (including depression under tertiary care) or metabolic disease (including insulin-controlled diabetes), stroke, contraindications to d-cycloserine (including alcoholism), epilepsy, claustrophobia, regular therapy with opioid analgesics, or oxygen therapy.

**TABLE 1:** Demographic information

**Participants**	91
**Median (range) age years**	70 (49–84)
**BMI kg·m^−2^**	27.1 (6.6)
**Smoking pack-years**	30 (27.4)
**Resting *S*_pO_2__ %**	94 (3)
**Resting heart rate beats·min^−1^**	81.1±12.7
**FEV_1_/FVC**	0.55±0.15
**FEV_1_ % pred**	58±21
**MRC**	3 (1)
**Age at onset years**	62±10
**Duration of breathlessness years**	8 (10.5)
**Total exacerbations n**	0 (2)
**Comorbidities**	
Reflux/heartburn	30
Asthma	30
Hypertension	27
Swelling of ankles	20
Surgery to the chest	12
Diabetes	12
Depression	10
Bronchiectasis	9
Heart attack	8
Osteoporosis	8
Peptic ulcer	8
Inflammatory bowel disease	7
Arrhythmia	6
Heart failure	4
Tuberculosis	3
Neuromuscular weakness	2

Data are presented as n, median (interquartile range) or mean±sd, unless otherwise stated. BMI: body mass index; *S*_pO_2__: peripheral blood oxygen saturation; FEV_1_: forced expiratory volume in 1 s; FVC: forced vital capacity; MRC: Medical Research Council.

### Behavioural measures

#### Self-report questionnaires

Building on our previous work [6, 7], we selected a set of questionnaires designed to probe the experience of living with COPD, focusing particularly on the potential influence of emotional and behavioural measures.

The following self-report questionnaires were completed and scored according to their respective manuals: Dyspnoea-12 (D12) questionnaire [35], Centre for Epidemiologic Studies Depression Scale [36], State-Trait Anxiety Inventory Trait [37], Fatigue Severity Scale [38], St George's Respiratory Questionnaire (SGRQ) [39], Medical Research Council (MRC) breathlessness scale [40], Mobility Inventory [41], Pittsburgh Sleep Quality Index [42], Catastrophic Thinking Scale in Asthma [43], and Pain Awareness and Vigilance Scale [44].

### Physiological measures

Spirometry and two modified shuttle walk tests (MSWTs) were collected using standard protocols [45, 46]. Participant height and weight were recorded.

### MRI measures

#### Image acquisition

Imaging was carried out using a 3 T MAGNETOM Trio (Siemens Healthcare, Malvern, PA, USA). A T1-weighted (MPRAGE) structural scan (voxel size 1×1×1 mm) was collected and used for registration purposes. A T2*-weighted, gradient echo planar image scan sequence (voxel size 3×3×3 mm) was used to collect functional imaging data during the word-cue and faces task.

### Word-cue task

Given sufficient repeated threatening exposures, the thought alone of a breathlessness-evoking situation such as climbing the stairs or having to hurry can prime brain networks that then exacerbate breathlessness itself. The resulting top-down cascade can even drive breathlessness perception in the absence of afferent inputs. We drew on this well-recognised link [47–49] to indirectly probe the brain's prior associations with breathlessness with a salient word-cue task [50]. Breathlessness-related word cues were selected to include a dynamic range of scenarios. In this instance, the presentation of breathlessness-related word cues can be considered as similar to a conditioned stimulus, triggering the brain networks associated with the real-life situation itself. During functional MRI (fMRI) scanning participants were shown a set of breathlessness-related word cues in white text on a black background for 7 s in a pseudo-randomised order. Participants were then asked “How breathless would this make you feel” (words-breathlessness (wB)) and “How anxious would this make you feel?” (words-anxiety (wA)). To each question participants responded within a 7-s window using a button box and visual analogue scale (VAS). The response marker always initially appeared at the centre of the scale with the anchors “Not at all” and “Very much” at either end. Before the scan session participants were given the opportunity to practice using the button box with a set of test words. A control condition consisting of a string of “XXXXXXXXXXXXXXX” with fixed length of 15 characters was presented four times over the course of the scan, each time for 7 s, and was used as a baseline measure of activity in response to the presentation of a visual stimulus. Any resulting significant activity could be ascribed to expectation-related processes triggered by the meaning of the words. No rating period followed these control blocks (supplementary figure S1). This analysis could be considered as similar to an expectation paradigm in which the brain activity relating to breathlessness-related word cues (controlled for visual effects of the non-words) represents the brain response to breathlessness expectation. The resulting brain activity patterns therefore provide a window into the processes of breathlessness perception and expectation.

### Faces task

In order to dissociate the brain's responses relating to breathlessness-specific anxiety from generalised anxiety we employed a second validated paradigm involving emotional faces [51]. Emotional facial expressions are widely recognised to activate the same brain pathways as the behavioural emotion conveyed by the expression itself [52]. Fearful facial expressions, for example, have been shown to correspond to activity within the amygdala, a region known to modulate fear processing. The speed and accuracy of task completion, which in this instance was the recognition of facial gender, under different emotional conditions can be used probe whether attention is affected by the emotion shown by the face. This draws upon work suggesting that the threat of breathlessness may absorb cognitive resources [53, 54]. Biased attentional processes, either towards or away from potentially threatening situations, may be mirrored by activity patterns within threat and fear brain networks. In this task, participants were shown human faces with either happy or fearful expressions (100% intensity) for 500 ms (drawn from the set described in [51]) in blocks of 30 s. A fixation cross was interspersed for 30 s between the blocks of faces. Reaction time and accuracy were recorded throughout the task. Further details pertaining to facial stimuli can be found within the supplementary material.

### Analysis

#### Behavioural analysis: factor identification

**FIGURE 2:**
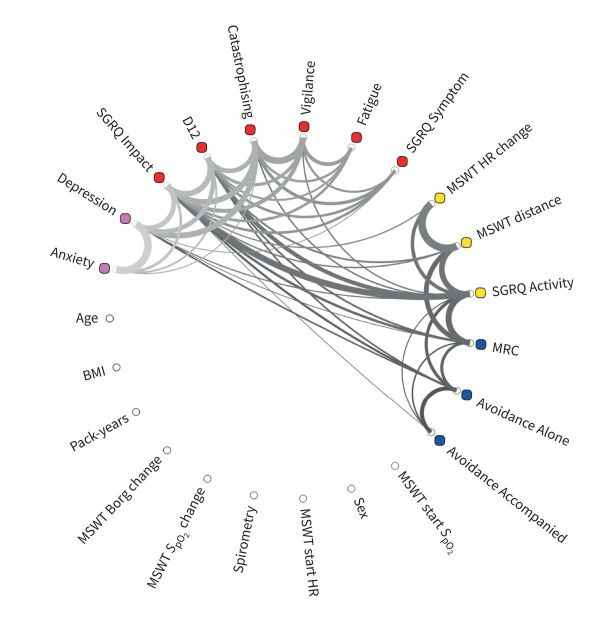
Connectogram: measures are plotted as a wheel with connections between each measure shown as a thread; the thickness of the thread provides a visual representation of the strength of the relationship between any two measures (r≥0.35). SGRQ: St George's Respiratory Questionnaire; D12: Dyspnoea-12; MSWT: modified shuttle walk test; HR: heart rate; MRC: Medical Research Council; *S*_pO_2__: peripheral blood oxygen saturation; BMI: body mass index. Coloured dots highlight the common factors underlying groups of measures. Pink dots correspond to Factor 4 (Mood), red to Factor 1 (Symptom burden), yellow to Factor 2 (Physical capability) and blue to Factor 3 (Perceived capability).

Full correlation matrices were calculated for (z-scored) behavioural questionnaires and physiological scores (spirometry, demographics and MSWT measures), using MATLAB 2017b (Mathworks, Natick, MA, USA). All included measures are shown in figure 2 and a list of these measures is included in the supplementary material. The structure of the correlation matrices was examined by applying a hierarchical cluster model to the data (supplementary figure S3). Hierarchical models use the covariance across groups of measures in order to organise them spatially within the correlation matrix [55–57]. The dataset was then visualised in figure 2 as a connectogram; a circular representation of interdependencies between measures. An exploratory factor analysis (EFA) was conducted to formalise these relationships [58–60]. EFA is a statistical method that uncovers latent (hidden) factors within a dataset.

#### Behavioural analysis: participant stratification

A principal component analysis was performed across the measures within each factor, identified *via* the EFA in the factor identification process. This information was input into the hierarchical cluster algorithm, alongside anonymised subject identities. A scree plot was used to identify the most statistically distinct groupings of participants (supplementary figure S6). The fMRI data was held out of the participant stratification process in order to provide independent validation of any resulting group differences at a later stage.

#### Imaging analysis

Image processing was carried out using the Oxford Centre for Functional Magnetic Resonance Imaging of Brain Software Library (FMRIB, Oxford, UK; FSL version 5.0.8; www.fmrib.ox.ac.uk/fsl), MATLAB (R2017b) and associated custom scripts. MRI processing was performed using FEAT (FMRI Expert Analysis Tool, within the FSL package).

#### Word-cue task lower-level analysis

At the individual subject level, a general linear model (GLM) was created with explanatory variables for breathlessness-related word or non-word presentation, and two de-meaned explanatory variables modelling the reported breathlessness (wB) and anxiety (wA) response to the word cues. The word cues represented a wide range of breathlessness-inducing situations. These ranged from very low impact activities such as quiet reading to high impact activities such as heavy lifting. Including ratings for each word in a first stage analysis allows for the variability between word cues to be incorporated into the model. An additional explanatory noise variable was included as a separate regressor to model the period during which the participant responded using the VAS. In addition to the mean contrasts for each of the explanatory variables, differential contrasts were also created for activity in response to breathlessness-related words greater than that for non-words and for non-words greater than that for breathlessness-related words (supplementary figure S2). It was this contrast of breathlessness-related words greater than for non-words that was of interest in the group-level analysis.

#### Faces task lower-level analysis

At the individual subject level, a GLM was created with explanatory variables for stimulus presentation periods of happy and fearful faces, along with the associated (de-meaned) reaction times. Two additional explanatory variables were created to model participant (de-meaned) accuracy in identifying whether the presented faces were male or female. In addition to the mean contrasts for each of the explanatory variables, differential contrasts were also created for activity in response to fearful faces greater than that for happy faces.

#### Group-level analysis

We then performed a cross-subject analysis to examine the expectation-related responses to breathlessness-related cues. This could be considered as similar to an anticipation paradigm in which chronic breathlessness participants are already primed to potential triggers of breathlessness. Mean voxel-wise differences in activity were calculated for the breathlessness-related words>non-words, non-words>breathlessness-related words. This allowed us to examine the brain activity resulting from automatic cued breathlessness perceptions generated by the breathlessness-related words. Contrasts were also created for fearful faces>happy faces, happy faces>fearful faces for the faces task. For both tasks, contrasts were also employed to examine the differences between the two groups of participants (corresponding to high and low symptom load) identified within the hierarchical cluster model at the participant stratification stage. In an additional analysis we asked the question “Does the relationship between expectation-related brain activity and word-cue ratings differ in our two behaviourally-defined groups?”. To answer this question, contrasts were also created between high and low symptom burden groups to examine brain activity that covaried with breathlessness anxiety (wA) and breathlessness intensity (wB) ratings. De-meaned age and sex values were modelled as regressors of no interest. Significance testing was performed using FSL's Randomise tool [61], which carries out rigorous permutation testing, with threshold-free cluster enhancement (TFCE) (a non-parametric test) [62] at family-wise error corrected p<0.05. Based on *a priori* hypotheses, a region of interest (ROI) approach, examining differential activity specifically within the amygdala (including 10-mm radius spherical masks based around a previously published peak voxels of a left amygdala region (−14/−6/−8) and its right hemisphere counterpart from an emotion regulation paradigm [63]), was also taken. This choice was driven by our previous work showing that this amygdala mask sensitively differentiates between patients with and without anxiety disorder [64, 65].

Supplementary analysis examined whether brain activity in response to happy or fearful faces differed between the two groups and is reported within the supplementary material.

## Results

Of the 23 variables entered into the hierarchical cluster model, EFA indicated that 14 should be retained for model validation. These variables included: Anxiety, Depression, SGRQ Impact, D12, Catastrophising, Vigilance, Fatigue, SGRQ Symptom, MSWT heart rate change, MSWT distance, SGRQ Activity, MRC, Avoidance Alone and Avoidance Accompanied. These groups of measures are demonstrated in figure 2 and in supplementary figure S3.

**FIGURE 3:**
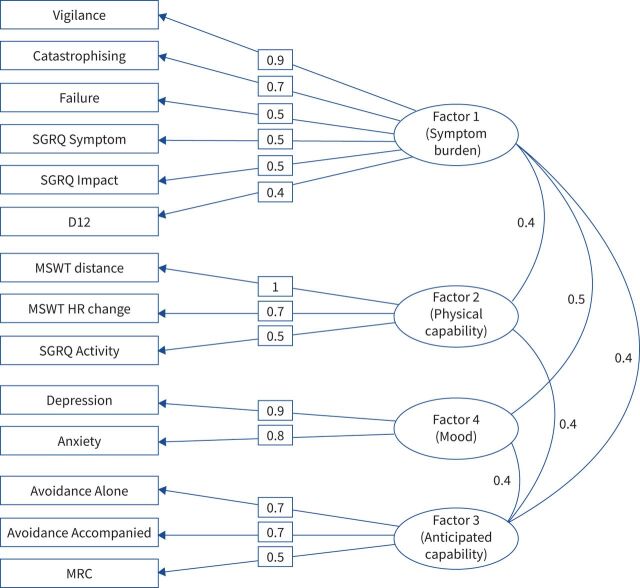
Factor loadings. SGRQ: St George's Respiratory Questionnaire; D12: Dyspnoea-12; MSWT: modified shuttle walk test; HR: heart rate; MRC: Medical Research Council. The relationship between each variable (rectangles) and its parent factor (ellipses) is shown. Loadings (straight lines) can be interpreted as correlation coefficients. The covariance between factors (curved lines) is also shown.

The composite scores of the 14 measures retained by the EFA formed four factors. This final four-factor model (shown overlaid onto figure 2) was validated after testing for models of two, three and four factors (χ^2^=65.85, df=41, p<0.008; Tucker–Lewis index 0.9; root mean square error of approximation 0.05). The factor diagram shown in figure 3 shows how four latent factors emerged from the 14 variables. Factor 1 is made up of Vigilance, Catastrophising, Fatigue, SGRQ Symptoms, SGRQ Impact and D12. Factor 2 is made up of MSWT distance, MSWT heart rate change and SGRQ Activity. Factor 3 is composed of two of the Avoidance subscales and the MRC scale. Finally, Factor 4 consists of Depression and Anxiety. The covariance between factors is illustrated by the curved lines in figure 3, with Factors 1 and 4 demonstrating the strongest covariance (0.5). This relatedness highlights that the factors should be considered as belonging to one model.

### Identifying phenotypes from the latent factors

**FIGURE 4:**
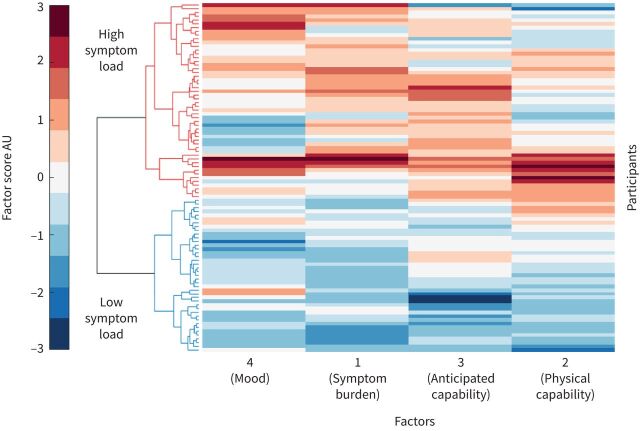
Clustergram: a matrix of each participants’ score across the four key factors identified by exploratory factor analysis. Factor score is measured in arbitrary units (AU). Participants form the *y*-axis, while each of the four factors is shown along the *x*-axis. A dendrogram is displayed along the left side, highlighting the division of subjects into two clear groups.

The factors identified by EFA model fitting were then used to stratify the patient population *via* their composite scores on these four major factors in a hierarchical cluster model (figure 4). A scree plot (supplementary figure S6) confirmed that a two-group solution was the most distinct. The groupings of participants corresponded to high symptom load and low symptom load across the four factors, with significant differences between the two groups between each factor at p<0.001. This difference was not driven by differences in spirometry scores (p=0.94).

### MRI results

#### Mean group differences: word task

**FIGURE 5:**
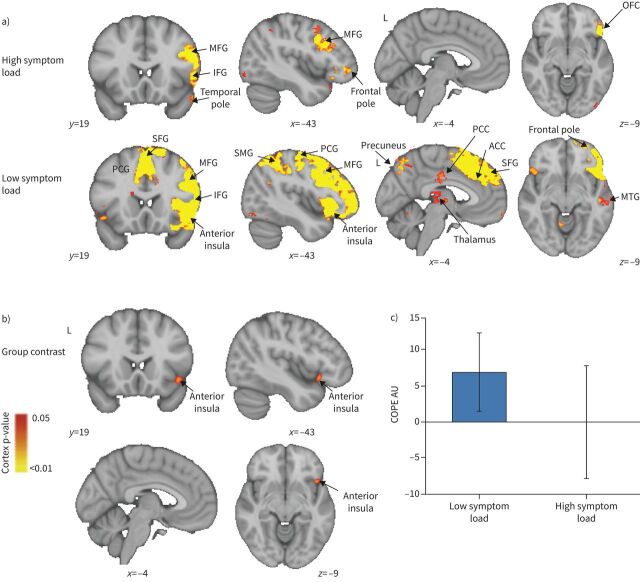
Blood oxygen level dependent activity in response to breathlessness-related words compared with non-words for a) high and low symptom load groups separately, and b) their contrast. MFG: middle frontal gyrus; IFG: inferior frontal gyrus; OFC: orbitofrontal cortex; PCG: paracingulate gyrus; SFG: superior frontal gyrus; SMG: supramarginal gyrus; PCC: posterior cingulate cortex; ACC: anterior cingulate cortex; MTG: middle temporal gyrus; COPE: contrast of parameter estimate. a) The IFG, MFG and temporal pole demonstrated significant activity in the high symptom group, while in the low symptom group, the PCG, anterior insular, temporal pole, precuneus, PCC, ACC, SMG, thalamus SFG, MFG and IFG all demonstrated significant activity. For both high and low symptom groups, significant regions are displayed with a non-parametric threshold-free cluster enhancement p<0.05. b) The contrast of the two groups revealed significant regional activity in the low symptom group compared with the high symptom group in the anterior insula cortex with a non-parametric p<0.05. c) Mean COPE in arbitrary units (AU) for the high and low symptom load groups separately with standard deviation bars showing the variation in parameter estimates across participants.

**FIGURE 6:**
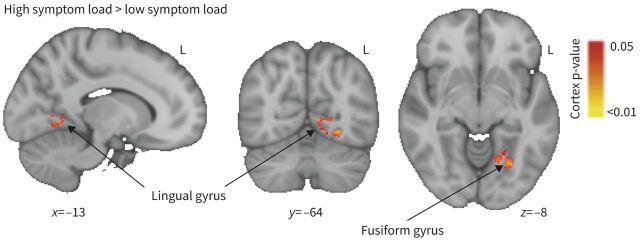
The positive correlation between blood oxygen level dependent activity and breathlessness ratings was stronger for the high symptom load group than low symptom load group in the lingual gyrus and fusiform gyrus. Significant regions are displayed with a non-parametric threshold-free cluster enhancement p<0.05.

In the word-cue task, which was designed to probe the brain's expectation-related response to breathlessness-related situations [50], significant group differences were found in response to the breathlessness-related word cues compared with non-words in the anterior insula (figure 5b) when using non-parametric permutation testing (TFCE) . The blood oxygen level dependent (BOLD) response within the low symptom group was found to be higher in this key region in response to breathlessness words *versus* non-words (TFCE p<0.05). This difference reflected the generally greater activity observed in the low symptom load group (6.97±5.42) compared with the high symptom load group (0.03±7.81) (figure 5a and c). No significant difference was observed within the small volume correction analysis of the amygdala. In an additional analysis we identified a stronger positive correlation between brain activity and ratings of breathlessness intensity (but not breathlessness anxiety) in the high symptom load group than the low symptom load group in the lingual/fusiform gyrus (figure 6).

**FIGURE 7:**
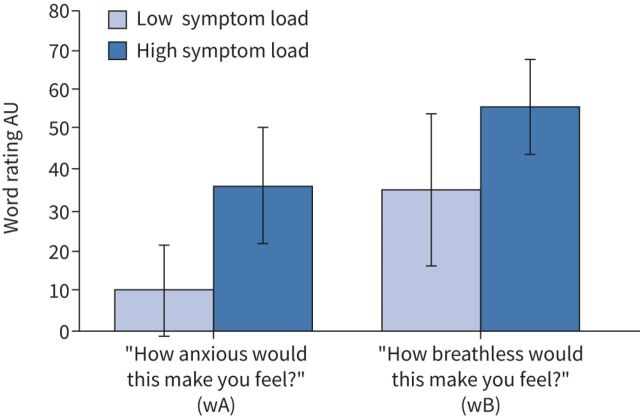
Participant ratings of breathlessness anxiety (words-anxiety (wA)) and breathlessness intensity (words-breathlessness (wB)) in response to breathlessness-related words for low and high symptom load groups. Data are presented as mean±sd for both groups.

Participant ratings of the breathlessness-related words were found to be significantly higher in the high symptom load group (p<0.01) compared with the low symptom load group for both breathlessness anxiety (wA) (high load 36.7±19.2, low load 10.3±11.2) and breathlessness intensity (wB) (high load 56.0±11.8, low load 35.4±14.8) (figure 7), both of which were measures that remained independent of the classification of the participants into groups.

#### Mean group differences: faces task

In the control task of emotional faces (to test for generalised anxiety), no significant difference in activity between groups in the condition of fearful *versus* happy faces was identified. No significant difference was observed within the small ROI analysis of the amygdala. No significant difference was found in participant reaction times to happy (high load 758.57±137.92 ms, low load 782.09±107.72 ms) or fearful faces (high load 742.71±141.87 ms, low load 773.70±125.71 ms) (p>0.05).

## Discussion

### Key findings

Using unsupervised machine learning we stratified a population of patients with COPD into two groups based on their scores across four latent (hidden) factors of breathlessness. We have described these factors as “Mood”, “Symptom burden”, “Anticipated capability” and “Physical capability”. Differences between the two groups were linked to expectation-related brain activity within the anterior insula, a region associated with the processing of bodily sensations and perceptions. These findings highlight the relevance of expectation-related brain processes to the lived experience of breathlessness, laying the foundation to therapeutically target brain processes and thereby address the individual's experience of breathlessness.

### A new mode of investigation for chronic breathlessness

Using a large sample size (91 participants) we were able to better understand variability in breathlessness perception than previously possible, where sample sizes were smaller. The data-driven analysis approaches employed offer an effective way to both discover and formalise new relationships within multimodal datasets such as this one without restricting or biasing the analysis.

In this study, the unsupervised machine learning algorithms indicated that 14 out of the 23 behavioural measures were important descriptors of the population. While each of these single measures may offer a partial description of breathlessness, a more powerful approach is to combine relevant measures and draw out common factors, thus uncovering data trends that may ordinarily be hidden by noise. In this case the four latent factors that were found to underly scores on these 14 measures together paint an intuitive picture of the lived experience of breathlessness: what a person feels they can and cannot do, how their symptoms impact their lives and their general mood.

Physiological measures such as spirometry and pack-years failed to fulfil the criteria of either contributing uniquely to a single factor or exceeding the required 0.4 weighting score to be considered as strongly loading onto a factor and therefore to be included in the model [60]. Furthermore, spirometry was not found to differ between the two groups. These findings highlight that the perception of breathlessness arises as an interplay between numerous factors, including physiology and the associated neural activity of expectation-related processes.

### Stratifying patients based on latent factors of breathlessness

The four factors produced by the EFA demonstrated significant intra-factor correlations (R=0.4–0.5) and so should be considered to belong to one model. By using participant scores across these four factors as inputs into the model-free technique of hierarchical clustering, we were able to examine how weightings across the factors differed across the patient population. The previously held-out fMRI data were then used to provide an external validation of the groupings and revealed that these differences appear to be linked to differences in the processing of breathlessness expectation cues. We identified greater activity in response to breathlessness-related word cues within the anterior insula for the low symptom burden group than the high symptom burden group.

Increases in insula activity are commonly reported in studies of induced air hunger [26–28], and in both the anticipation and application of resistive loads [8, 66–68]. In these studies, insula activity has been found to be higher in conditions of breathlessness or the anticipation of breathlessness than at baseline. However, in the current study the lower symptom load group demonstrated greater activity in the anterior insula. This result implies that a greater level of breathlessness symptom load does not necessarily equate to a proportionally greater level of associated brain activity and that neural encoding might not be reflected in a simple linear stimulus–response relationship. An alternative explanation draws on recent work that is beginning to discuss brain function in terms of networks [69–71], rather than tying specific functions to particular brain areas [72–74]. Brain regions are now thought to subserve many different functions through these network interactions, including bodily awareness (interoception) [74, 75].

The anterior insula is part of the wider brain network thought to be involved with the affective perception of internal sensations and the evaluation of stimuli [76, 77], and has itself been linked with the processing of subjective feelings [78]. Therefore, the greater activity within the network of the low symptom load group may be linked to a more coherent representation of internal sensations (*i.e.* Factors 2 and 3), leading to lower anxiety and general symptom burden (*i.e.* scores on Factors 4 and 1). While for the high-burden group, breathlessness cues may trigger more complex and personal internal representations, resulting in greater individual variation in brain activity patterns. This hypothesis is in part supported by our findings of a stronger correlation between activity in the lingual/fusiform gyrus and breathlessness intensity ratings in the high symptom load group. The lingual/fusiform gyrus resides with the ventral visual processing stream, a network of regions with well-established specialism for faces, words and objects [79–81]. *Via* reciprocal connections with both lower visual and higher cortical areas, the lingual/fusiform gyrus combines lower-level visual features to extract semantic meaning [82]. In the case of words, activity within the lingual/fusiform gyrus has been shown to be influenced by emotional content [83], increasing with negative valence [84]. While there is some evidence to suggest that the lingual gyrus may be connected to fear- and anxiety-related processes [85, 86], other work has highlighted that greater cognitive demands and focused attention can also increase brain activity within a “motivated attentional framework” [83, 87, 88].

In this study we demonstrated a stronger positive correlation with breathlessness intensity ratings and brain activity for the high, compared with low, symptom load group within the lingual/fusiform gyrus. This finding may reflect a positive relationship between word-cue intensity and attentional demands or one in which the high symptom load group is more primed by their own previous experiences to increasing negative salience of the word cues. Either of the cases (which are also not mutually exclusive) could result in a greater positive correlation between regional brain activity and ratings of breathlessness intensity in response to the word cue.

Additionally, it is worth noting that the involvement of the lingual/fusiform gyrus may be directly related to the task (in this case word cues). Another type of cue, *e.g.* a breathing load or auditory stimulus, would act *via* different mechanisms and activate other brain areas. In contrast, the insula takes input, among others, from sensory processing brain regions. Therefore, the differences in activity observed within the insula may represent a more “downstream” and therefore more stimulus-independent group difference in the affective processing of breathlessness expectation.

### Further considerations and limitations

The techniques employed here draw upon the big data techniques of machine learning and with further participants it may be possible to gain sufficient statistical power to further probe subgroups, such as those visible within figure 4. Such groupings could of course be considered as existing as part of a broader distribution, where people fall onto a spectrum of symptoms. However, providing clear boundaries in this case enables us to initially consider the differences between people, which may relate to viable treatment options. Another important caveat of cluster methods is that they examine shared variance and so we must remain aware that any of the measures not included in this model could be highly relevant, but not share any common features with the other measures. Additionally, in this cross-sectional work, the direction of causality is impossible to infer: are people with low mood more susceptible to breathlessness anxiety or do they become low in mood as a result of their greater symptom burden? Likewise, when considering links between the latent factors and brain activity patterns, is high symptom burden a result of aberrant brain processes or a driver of the patterns themselves? Longitudinal population studies would thus need to be employed to understand any predominating direction within these relationships. Future studies could also consider using resting state brain activity to examine the network dynamics underlying this difference. Finally, we suggest that future work should include both detailed physiological and behavioural measures, which together could better characterise breathlessness and unexplained variance in patient-reported outcomes.

This work opens up new avenues for improving patient treatment outcomes. First, the models developed here could be tested for their ability to predict treatment outcomes. If all or some of the model factors were shown to be therapeutically relevant, then their modulation could become the focus of targeted treatment programmes. Second, the differences in brain activity patterns between the two groups could represent a therapeutically relevant difference in breathlessness processing. The concept of “fooling the brain to treat the lungs”, coined by Similowski [89], highlights the importance of considering the brain as a target for the treatment of breathlessness. In this instance, linking brain activity to therapeutic differences *via* latent factors of breathlessness as demonstrated here is advantageous when considering the cost and restricted applicability in the wider population of MRI scanning.

### Conclusions

By combining neuroimaging, behavioural and clinical measures we have created an enriched model that enables us to examine breathlessness from a multimodal perspective. In so doing we were able to stratify participants, linking group differences in symptom burden to expectation-related brain activity within the anterior insula, an area well recognised for processing breathlessness as well as internal bodily perceptions. These findings potentially move us towards therapeutically relevant neuro-biomarkers and tailored treatment programmes, which may together address the lived experience of breathlessness within an individual.

## Supplementary material

10.1183/13993003.04099-2020.Supp1 **Please note:** supplementary material is not edited by the Editorial Office, and is uploaded as it has been supplied by the author.
Supplementary material ERJ-04099-2020.SUPPLEMENT

## Shareable PDF

10.1183/13993003.04099-2020.Shareable1 This one-page PDF can be shared freely online.
Shareable PDF ERJ-04099-2020.Shareable
