# Supplementary material for: Breathlessness in COPD: linking symptom clusters with brain activity
Source: Eur Respir J. 2021 Nov 18;58(5):2004099. doi: 10.1183/13993003.04099-2020 (PMC8607925; doi:10.1183/13993003.04099-2020)
Supplement: Supplementary file 1 [file ERJ-04099-2020.SUPPLEMENT.pdf]

Supplementary Material

Breathlessness in COPD: linking symptom clusters with brain activity

Sarah L. Finnegan (sarah.finnegan@ndcn.ox.ac.uk)

Olivia K. Harrison (faull@biomed.ee.ethz.ch)

Catherine J. Harmer,

Mari Herigstad,

Najib M. Rahman,

Andrea Reinecke,

Kyle T.S. Pattinson (kyle.pattinson@nda.ox.ac.uk)

Online Data Supplement

Disclosure

This body of work was collected as part of a larger project investigating the effect of D-cycloserine (a partial NMDA receptor agonist, with target sites that include the amygdala) on outcome measures of pulmonary rehabilitation. This population forms the baseline component of this study and full results of the trial will be published separately and at a later date.

Behavioural Measures

Questionnaire Measures

Dyspnoea-12 (D12) Questionnaire: This is a 12-item questionnaire designed to measure the severity of breathlessness and has been validated for use in patients with respiratory disease [1].

Centre for Epidemiologic Studies Depression Scale (CES-D): Depressive symptoms are commonly observed in patients with respiratory disease. This brief questionnaire consists of 20 items investigates the symptoms of depression across a number of factors [2].

State - Trait Anxiety Inventory - Trait (STAI-T): This questionnaire assesses participant's general level of anxiety in particular scenarios via 20 questions asking "how anxious you generally feel" [3].

Fatigue Severity Scale: This 9-point questionnaire quantifies patient fatigue, which is well documented in its association with COPD [4].

St George's Respiratory Questionnaire (SGRQ): There are 50 questions in this questionnaire, which has been developed and validated for use in COPD and asthma. The questions measure the impact of overall health, daily life and well-being [5].

Medical Research Council (MRC) breathlessness scale: The MRC scale quantifies perceived difficulty due to respiratory restrictions on a scale of 1 to 5 [6].

Mobility Inventory (MI): This questionnaire collects data regarding the extent to which a participant avoids certain situations, either alone or accompanied (21-items in each category) [7].

Catastrophic Thinking Scale in Asthma: This 13-point questionnaire was modified for this study by substituting the word "asthma" for "breathlessness" in order to measure catastrophic thinking [8, 9].

Pain Awareness and Vigilance Scale: This questionnaire was modified by substituting the word "breathlessness" for the word "pain". The 16-point scale measures how much a participant focuses their attention onto their breathlessness [9, 10].

Physiological Measures

A trained respiratory nurse collected spirometry measures of FEV_1 and FVC using Association for Respiratory Technology and Physiology standards [11]. Participants performed two incremental shuttle walk tests (MSWT) [12], and heart rate and oxygen saturations (SpO_2) were measured immediately before the MSWT and subsequently every minute until 10 minutes post-exercise (or until participants returned to their baseline state) using a fingertip pulse oximeter (Go_2; Nonin Medical Inc). Before and after the MWST participants also rated their breathlessness on a modified Borg scale [13]. In a MWST participants must walk between and around two cones, placed 10m apart in time to a set of auditory beeps played from a laptop. Initially the speed of beep repetition is slow, but the participant must increase their walking speed each minute in order to reach the cone before the next beep. Participants continue to walk (or run) until they are too breathless to continue, at which point the total distance walked is recorded.

Imaging Measures

Word Task

This task was developed and published by Herigstad and colleagues in 2016 for use in the COPD population [14]. Word cues were developed in three key stages; firstly in collaboration with respiratory practitioners, academics and physiotherapists, a set of 30 word cues associated with breathlessness were created. Next, these cues were provided to patients with COPD alongside a VAS rating scale, allowing patients to rate

how breathless and anxious the situations identified by the cues would make them feel. Following adjustments based on participant feedback, the word cues were then computerised and tested in a larger population of COPD patients [14]. Further validation was carried out in the fMRI environment and by for clinical sensitivity with comparisons between changes in key questionnaire measures and word-cue rating.

Faces Task

Faces were drawn from a set first developed by Ekman and Friesen [15] and furthered by Young et al [16]. Photographs of 10 faces (5 male, 5 female) with fearful or happy expressions of 100% intensity were used. Each face was shown for 500ms in blocks of 30 seconds. A fixation cross was interspersed for 30 seconds between the blocks of faces. Participants were instructed to respond via a button box to indicate facial gender. Reaction time and accuracy were recorded throughout the task. The task contrasting fear and happy facial expressions has been extensively used in previously studies and has been found to activate the amygdala in both healthy volunteers and in depressed patients [17, 18]. Neutral faces are not typically used as they can be interpreted as threatening or ambiguous in different settings [19].

MRI Acquisition

Prior to each MRI session participants were screened for standard MRI contraindications including metal in or about their person, epilepsy and claustrophobia.

Sequence Parameters

Hardware: A Tim System (Siemens Healthcare GmbH) 12-channel head coil.

T1 sequence parameters: TR, 2040ms; TE, 4.68ms; voxel size, 1 x 1 x 1 mm; FOV, 200mm; flip angle, 8°; inversion time, 900ms; bandwidth 130 Hz/Px).

T2*-weighted (functional) sequence parameters: TR, 3000ms; TE 30ms; voxel size 3 x 3 x 3 mm; FOV, 192mm; flip angle 87°; echo spacing 0.49ms.

Functional scan durations: word-task - 215 volumes, 10 minutes and 27 seconds duration and faces task - 168 volumes, 8 minutes and 24 seconds duration.

Field map scans of the B_0 field were obtained to aid the distortion correction of the functional scans: TR, 488ms; TE1, 5.19ms; TE2, 7.65ms; flip angle 60°; voxel size, 3.5 x 3.5 x 3.5 mm.

Analysis

Cluster Analysis

For this analysis, only data from participants (n=91) who completed both the behavioural and MRI parts of the study were utilised. When calculating the full correlation matrices for the hierarchical clustergrams, scoring was reversed for the following measures: MSWT Distance, MSWT starting oxygen saturation, MSWT heart rate change and spirometry. This ensured that higher scores were associated with worse symptomatology across all measures.

The full list of measures included in the cluster analysis is as follows:

1. State-Trait Anxiety Inventory (STAI-T) – Trait – Measuring Anxiety
2. Centre for Epidemiologic Studies Depression Scale (CES-D) – Measuring Depression
3. St George's Respiratory Questionnaire (SGRQ) – Impact Domain
4. Dyspnea-12 Questionnaire (D12)
5. Catastrophic Thinking Scale in Asthma – Measuring Catastrophising
6. Pain Awareness and Vigilance Scale – Measuring Vigilance
7. Fatigue Severity Scale – Measuring Fatigue
8. St George's Respiratory Questionnaire (SGRQ) – Symptom Domain
9. Modified Shuttle Walk Test (MWST) – Heat Rate Change

10. Modified Shuttle Walk Test (MWST) – Distance
11. St George's Respiratory Questionnaire (SGRQ) – Activity Domain
12. Medical Research Council (MRC) breathlessness scale
13. Mobility Inventory (MI) – Measuring Avoidance Alone
14. Mobility Inventory (MI) – Measuring Avoidance Accompanied
15. Modified Shuttle Walk Test (MWST) – Starting Oxygen Saturation
16. Sex
17. Modified Shuttle Walk Test (MWST) – Starting Heart Rate
18. Spirometry
19. Modified Shuttle Walk Test (MWST) – Oxygen Saturation Change
20. Modified Shuttle Walk Test (MWST) – BORG Change
21. Smoking Pack Years
22. Age
23. Body Mass Index

Hierarchical cluster models reorder variables based on their correlation strengths so that groups of related measures sit closer to each other than non-related measures. This allows natural relationships to be easily visualised [20-22]. The modelling process formalises not only the relationship between pairs of variables, but also the manner by which shared variance can be described as part of larger, related clusters. The clustering algorithm initially considers pairs of variables in terms of their similarity or "distance" (in arbitrary units). Linked pairs are then incorporated into larger clusters with the goal of minimizing a cost function (distance to be bridged), a process that can be thought of as minimizing the dissimilarity within clusters. As pairs become clusters, a cluster tree or dendrogram is created. The distance between neighbouring branches indicates the relative similarity of two measures, while advancing up the hierarchical cluster tree moves further away in terms of link distance, and therefore similarity.

Hierarchical models are useful as a descriptive tool for examining and visualising the structure of the dataset as a whole. However, they do not provide information as to the significance of any given cluster of behavioural measures. In contrast, exploratory factor analysis (EFA), which falls under the umbrella of structural equation modelling, can be used to formalise the relationships observed in the hierarchical models [23-25]. This allows the researcher to establish the presence of underlying shared constructs via a number of fit statistics without applying a preconceived structure on the result.

For the EFA analysis, the smallest number of uncorrelated clusters that maximally explain the variance of the dataset was estimated. In this instance, parallel analysis with oblique rotation was employed to calculate this value and the results were visualised using a scree plot (Supplementary Figure S5). In a second step, the number of variables to be retained within the model was determined. A maximum likelihood estimation approach was applied, where variables that did not load significantly onto a particular factor, or demonstrated significant cross loading (i.e. loaded onto more than one factor) were excluded from further testing. Finally, the model statistics were interrogated to formalize the shared variance across latent factors and the extent to which each variable contributed to its factor as a whole. The smallest number of factors that significantly explained the variance across the dataset was then accepted as the model of best fit. Model selection criteria included loading variables above 0.4 with no cross loading or freestanding variables, and significant X^2/df ratio with Tucker-Lewis Index (TL-index) close to 1 and RMSEA < 0.06. Models were fit using Lavaan version 0.6-1 [26] in R version 3.2.1 (R Core Team).

Imaging Analysis

MRI Preprocessing

The data were corrected for movement using MCFLIRT (Motion correction using FMRIB's Linear Image Registration Tool [27]). Non-brain structures were removed

using BET (Brain Extraction Tool [28]). Spatial smoothing was carried out using a full-width-half-maximum Gaussian kernel of 5mm, while high-pass temporal filtering (Gaussian-weighted least squares straight line fitting; 90 s) removed low frequency noise and slow-drift.

Distortion correct of EPI data was carried out using a combination of FUGUE (FMRIB's Utility for Geometrically Unwarping EPI's [29, 30] and BBR (Boundary Based Registration; part of the FMR Expert Analysis Tool, FEAT version 6.0 [31]).

Data denoising was carried out as follows: Before the first level analysis, each functional scan was decomposed into maximally independent components using FMRIB's MELODIC tool (Multivariate Exploratory Linear Optimised Decomposition into Independent Components). "Noise" components were identified by FIX (FMRIB's auto-classification tool, [32, 33]) using the WhII.Standard.RData [34] trained classifier with aggressive clean up option. A Principle Component Analysis (PCA) was run on the FIX identified components to retrain 99% of the variance. Separately, the cardiac and respiratory related physiological signals (recorded via a pulse oximeter and a respiratory bellows) were transformed into a series of regressors, (three cardiac and four respiratory harmonics) as well as an interaction term and a measure of respiratory volume per unit of time (RVT), using FSL's physiological noise modeling tool (PNM). The signal associated with these waveforms (modeled using retrospective image correction (RETROICOR) [35, 36]) was then used to form voxelwise noise regressors.

The confounds identified by FSL's FIX and PNM tools, along with sources of noise arising from motion, were then combined into a single model. This single noise model approach builds upon the technique outlined by [37]; and fully detailed by [38]. In these preceding works we employed a step-wise technique whereby physiological noise (identified by PNM) and FIX-identified noise were each removed from the data in

separate steps prior to data entry into the lower level model. In the new cleanup pipeline, a single text file containing time-course information relating to FIX identified noise components along with white matter or CSF related noise was included as additional confound EV's within the lower level model, while the PNM-identified noise was entered into the model as a standard voxel-wise confound list. In this updated de-noising pipeline, confounds identified above are added to model at the stage of first-level analysis and thus the functional dataset can be corrected for sources of noise arising from motion, scanner and cerebro-spinal fluid artefacts, cardiac, and respiratory noise in a single step, rather than three.

Image Registration

The functional scans were registered in a two-step process to the MNI152 (1x1x1 mm) standard space brain template. Firstly, each subject's EPI was registered to their associated T1-weighted structural image using BBR (6 DOF) with nonlinear field map distortion correction [31]. In the second step the subject's structural image was registered to 1mm standard space via an affine transformation followed by nonlinear registration (using FNIRT: FMRIB's Non-linear Registration Tool [39]).

Functional MRI Analysis

MRI processing was performed using FEAT (FMRI Expert Analysis Tool within the FSL package)

Word Task

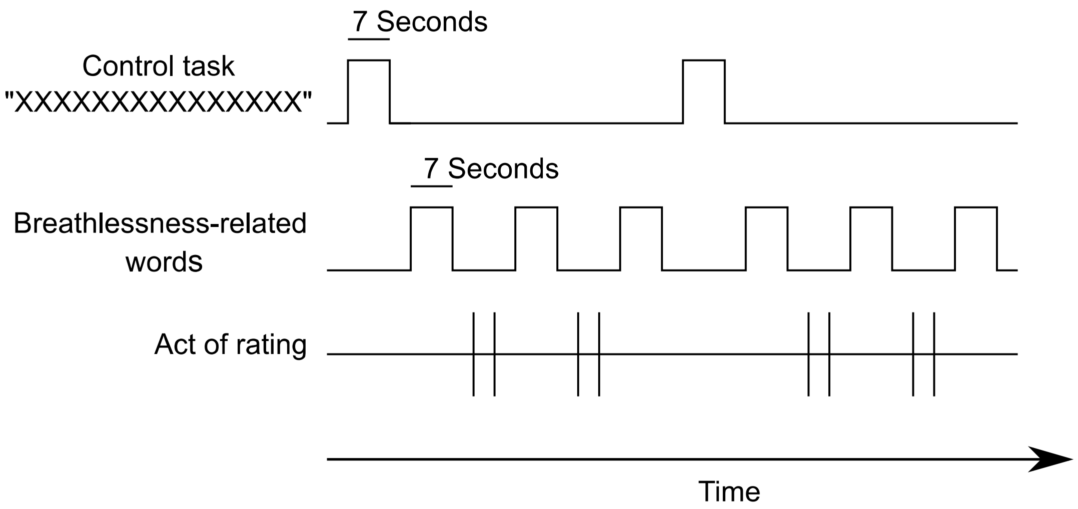

Supplementary Figure S1 Design schematic illustrating a sample sequence of breathlessness-related words, non-words and act of rating. The model for each individual contained: (1) 7 second blocks of breathlessness-related word cue presentation, (2) 7 second blocks of non-word cue presentation (no rating period followed these cues), (3) the rating scores of breathlessness related anxiety (wA) and breathlessness (wB). These scores were de-meaned. (4) The 7 second window of the rating period, which occurred 1 second after the cue presentation. This was included to account for the act of rating.

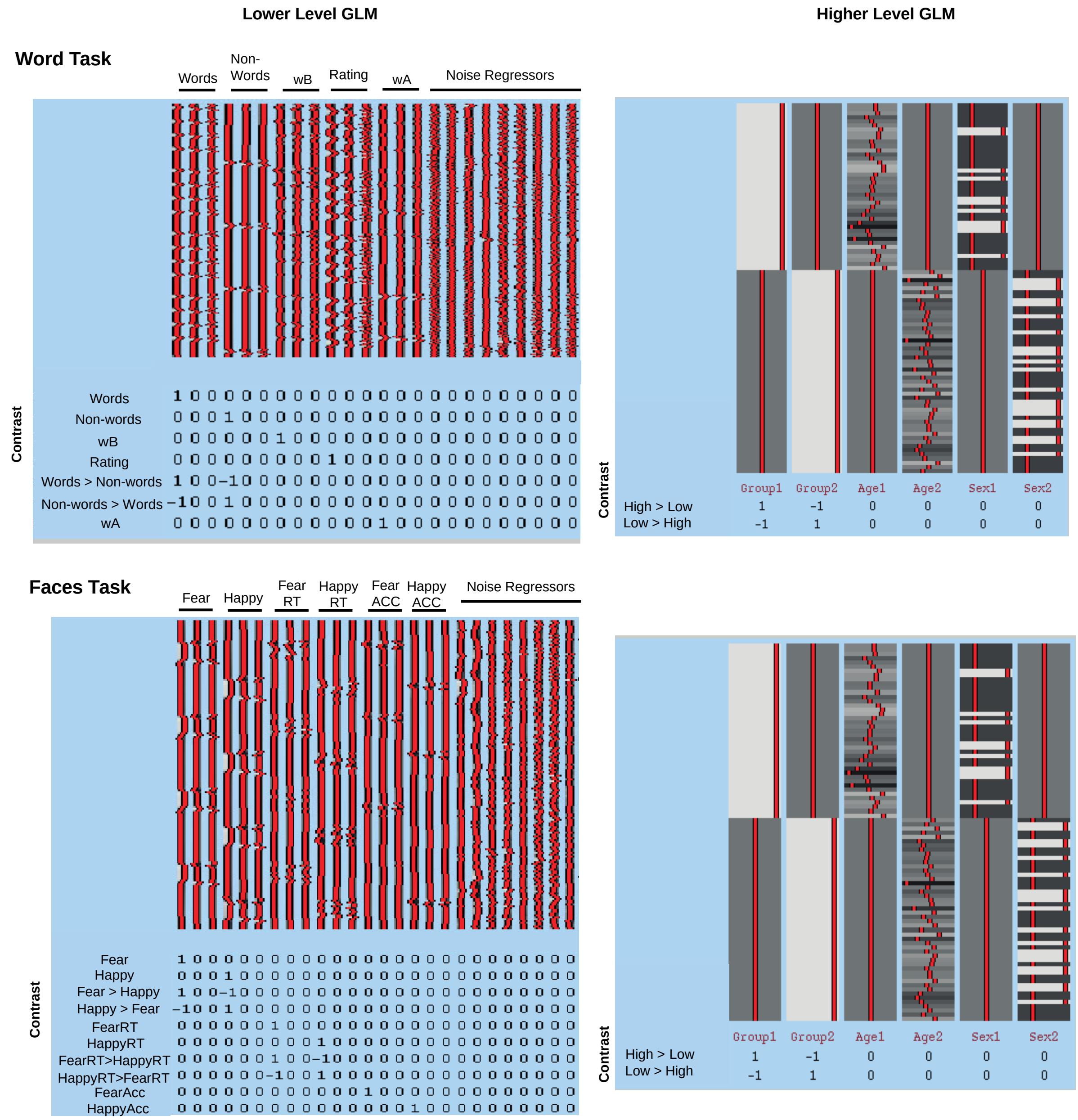

Supplementary Figure S2 – An illustration of the generalised linear models (GLM) used for both lower and higher level analyses for the word and faces task. Abbreviations as follows – wB – breathlessness rating, wA – breathlessness anxiety rating, RT – reaction time, ACC – accuracy. High and Low correspond to high and low symptom load respectively.

Faces Task

To examine whether brain activity differed between the two behaviourally identified

groups in the amygdala, a single logistic regression model was carried out using MATLAB's mnrfit function. Group identity was set as the independent variable, while mean brain activity within the amygdala in response to fearful faces, happy faces and their contrasts (fearful > happy / happy > fearful) were considered as dependent variables. Significance within the logistic regression was set at $p<0.05$, and values are reported as FDR corrected. To examine whether happy or fearful faces significantly activated the amygdala across both groups, t-tests were carried out for each contrast. Significance was set at $p<0.05$, FDR corrected.

Results

Supplementary Table S1 – Demographic information (N=100) recorded as either median and interquartile range (IQR) or mean and standard deviation (SD). Abbreviations (BMI – Body Mass Index, SpO_2 – peripheral oxygen saturation, FEV1 – Forced Expiratory Volume in one second, FVC – Forced Vital Capacity, MRC – Medical Research Council scale).

Age (median years/range)	**70 / (49-84)**
BMI kg.m-2 (median / IQR)	**27.3 (6.8)**
Smoking pack-years (median / IQR)	**30 (29.4)**
Resting SpO_2% (median / IQR)	**95 (3)**
Resting heart rate beats.min-1 ± SD	**80.8 ± 13.6**
FEV1/FVC ± SD	**0.55 ± 0.15**
FEV1% Predicted ± SD	**58 ± 21**
MRC (IQR)	**3 (1)**
Age of onset ± SD	**61.3 ± 10**
Duration of breathlessness (median years / IQR)	**8 (10.5)**
Total exacerbations (median number / IQR)	**0 (2)**

Comorbidities (frequency)		
Reflux/ heart burn (32)	Asthma (32)	Hypertension (31)
Swelling of ankles (25)	Surgery to the chest (13)	Diabetes (13)

Depression (13)	Bronchiectasis (9)	Heart attack (9)
Osteoporosis (8)	Peptic ulcer (8)	Inflammatory bowel disease (7)
Arrhythmia (6)	Heart failure (4)	Tuberculosis (3)
Neuromuscular weakness (2)		

Supplementary Table S2. Average participant psychological scores recorded as mean and standard deviation

Anxiety (39.2 ± 9.4)	D12 (12.6 ± 8.1)
Catastrophising (12.2 ± 10.2)	Vigilance (37.7 ± 15.7)
Depression (14.4 ± 8.6)	Fatigue (38.9 ± 12.9)
Avoidance – Alone (1.7 ± 0.7)	Avoidance – Accompanied (1.5 ± 0.5)
St George – Impact (34.5 ± 16.1)	St George – Symptom (63.4 ± 18.6)
St George – Activity (65.2 ± 20.88)	

Supplementary Table S3. Average participant scores for breathlessness related anxiety (wA) and breathlessness (wB) during the scanner word task. Recorded as mean and standard deviation, significance is recorded as **p<0.001

Mean wA	Mean wB
24.5 ± 20.66**	46.5 ± 16.7 **

The value of combining EFA with a hierarchical cluster model can be visualised by comparing Supplementary Figure S4 with Figure 4. In Supplementary Figure S4 all available behavioural measures have been included in a hierarchical cluster model.

As a result, while a colour gradient top-bottom (red to blue respectively) is roughly visible, no clear structure can be seen in the dendrogram shown left of the figure. In contrast, once the number of dimensions have been reduced, as shown in Figure 4, clear groups can be observed in the dendrogram – shown left of the central correlation matrix

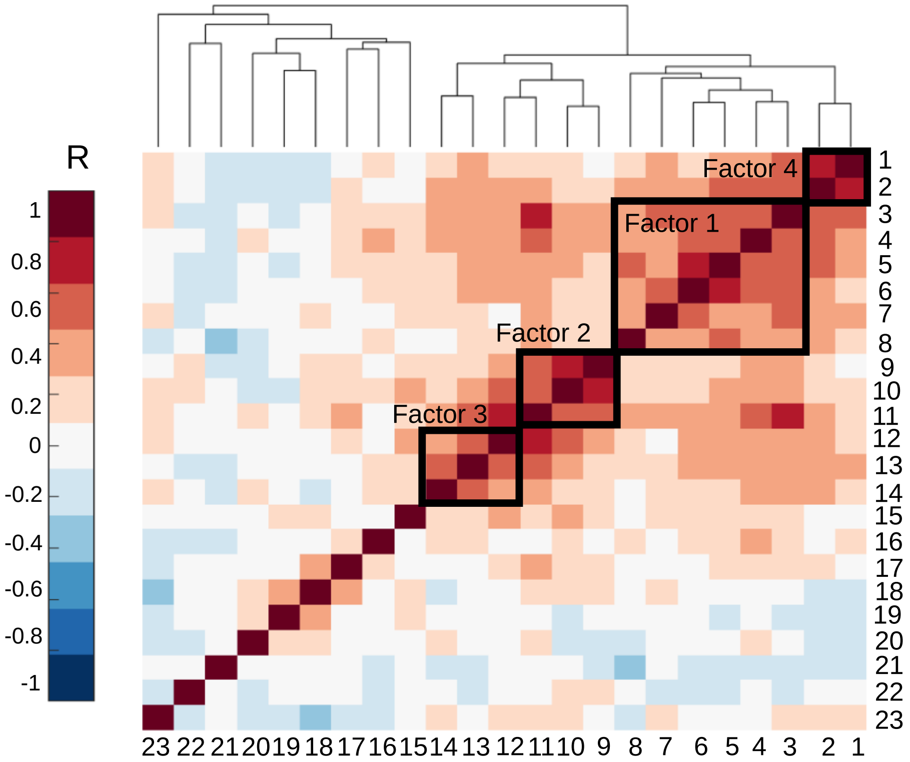

1	Anxiety	9	MSWT HR Change	17	MSWT Start HR
2	Depression	10	MSWT Distance	18	Spirometry
3	St George - Impact	11	St George - Activity	19	MSWT SATS Change
4	D12	12	MRC	20	MSWT BORG Change
5	Catastrophising	13	Avoidance - Alone	21	Pack Years
6	Vigilance	14	Avoidance - Accompanied	22	Age
7	Fatigue	15	MSWT Start SATS	23	BMI
8	St George - Symptom	16	Sex		

Supplementary Figure S3. Clustergram: a correlation matrix of measured behavioural and physical variables, where strength of the correlation is measured as a Pearsons' R-

value. Variables are reordered such that more closely related measures are placed proximal to each other. The relationship between groups of measures is demonstrated by the height of the dendrogram branches and distance between neighbouring branches (in arbitrary units). Clusters identified by the EFA as significant are highlighted by black boxes. Abbreviations are as follows: MSWT – Modified Shuttle Walk Test, SATS – Blood Oxygenation saturation, HR – Heart Rate, BMI – Body Mass Index

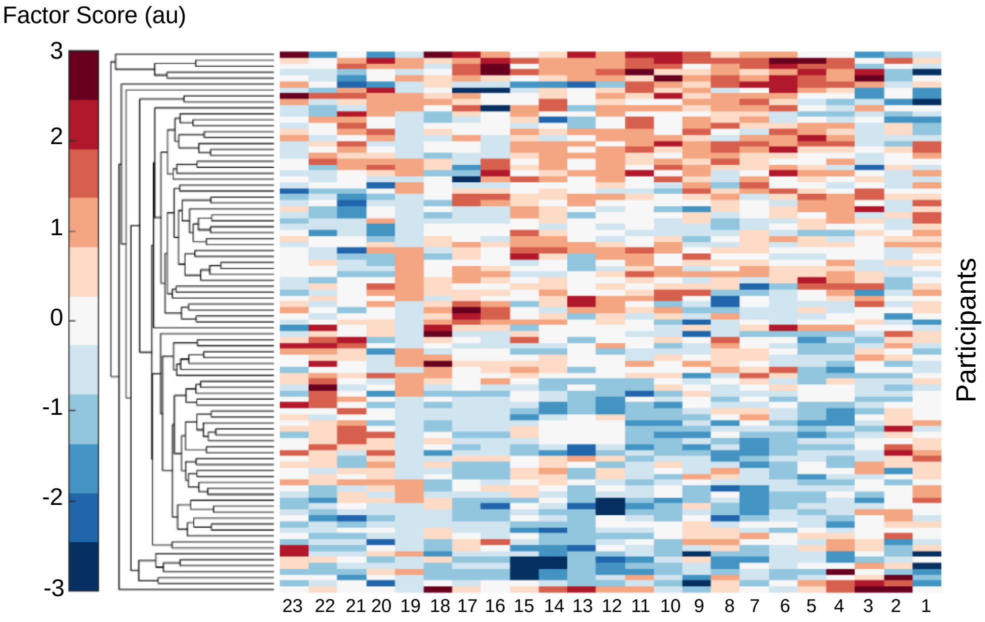

1 Age	9 St George - Symptom	17 Avoidance - Accompanied
2 Pack Years	10 D12	18 MSWT Start SATS
3 BMI	11 St George - Impact	19 Sex
4 Anxiety	12 St George - Activity	20 MSWT Start HR
5 Depression	13 MRC	21 Spirometry
6 Catastrophising	14 MSWT Distance	22 MSWT SATS Change
7 Vigilance	15 MSWT HR Change	23MSWT BORG Change
8 Fatigue	16 Avoidance - Alone	

Supplementary Figure S4. Clustergram: a matrix of each participants score across the all measures (shown within the table). Factor score is measured in arbitrary units (au). Participants form the y-axis, while each variable is shown along the bottom. A dendrogram is displayed along the left side with no clear subject grouping. Abbreviations are as follows: MSWT – Modified Shuttle Walk Test, SATS – Blood Oxygenation saturation, HR – Heart Rate, BMI – Body Mass Index

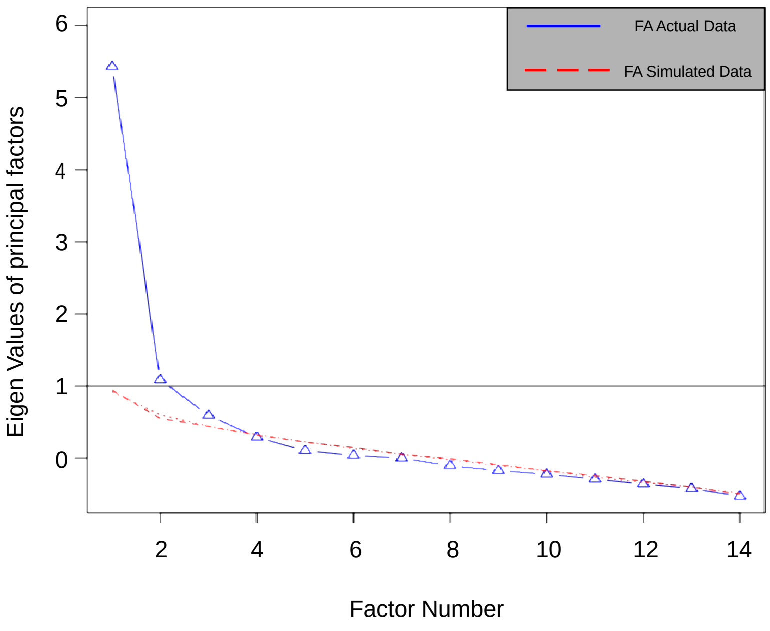

Supplementary Figure S5. **Scree plot – Assists visually in determining how many separate factors are present as part of the process of exploratory factor analysis. Created using parallel analysis, where the eigenvalues of the real data (blue solid line) are shown for each factor extracted compared to extracting the same number of factors from a similarly sized random dataset (red dotted line). Eigenvalues are shown on the y-axis, while factor number is plotted on the x-axis. The point at which the Eigenvalues of the real dataset are no longer greater than that drawn from the random dataset indicates the most distinct number of factors (i.e. where the red line crosses the blue line).**

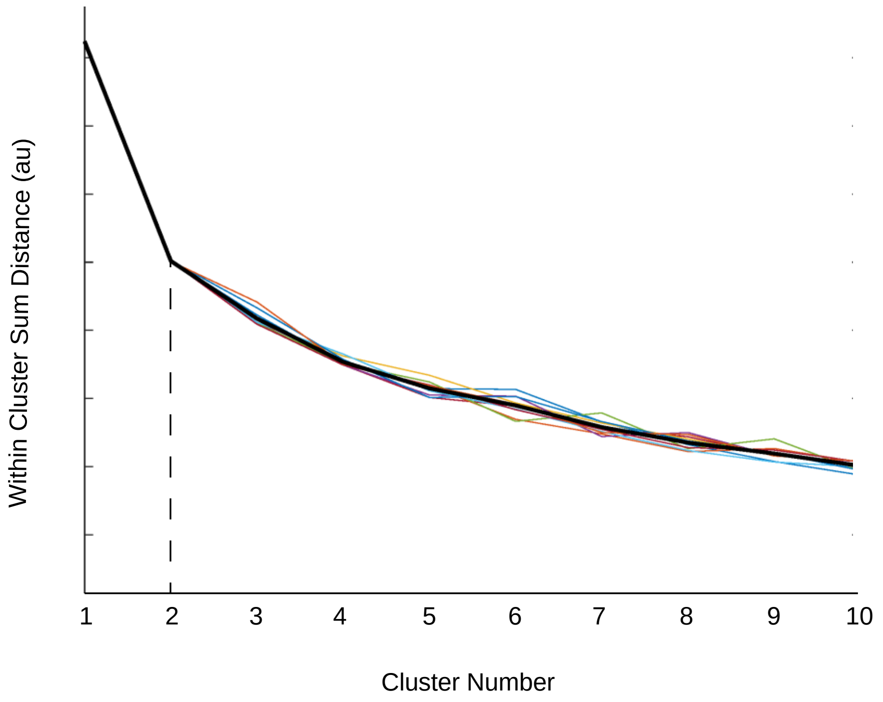

Supplementary Figure S6. Scree plot – Assists in a visual inspection of the most distinct number of participant clusters. Where within cluster distance (y-axis, au –arbitrary units) is plotted as a function of cluster number (x-axis). The point at which the addition of further clusters no longer significantly explains more of the dataset variance can be visualized as an "elbow" in the plot. The process is run several times and each of the coloured lines represents a single trial. The thicker black line represents the average of all trials (N=10). The dashed line highlights the elbow point of the graph, in this instance at 2 clusters.

Supplementary Table S4 – Group differences, determined by hierarchical cluster modelling, across key physiological and demographic measures (N=91) – recorded as either median and interquartile range (IQR) or mean and standard deviation (SD) depending on the distribution of the data. Abbreviations (BMI – Body Mass Index, FEV1 – Forced Expiratory Volume in one second, SpO_2 – peripheral oxygen saturation. (p values uncorrected).

	**High Load**	**Low Load**	**Difference**
Age (median years/range)	**71 / (49-83)**	**71 / (49-84)**	**p=0.22**
BMI kg.m-2 (median / IQR)	**26.8 (4.8)**	**27.6 (8.2)**	**p=0.25**
Smoking pack-years (median / IQR)	**40 (36.5)**	**28.8 (21.3)**	**p=0.04**
FEV1 % Predicted ± SD	**60.6 ± 19.3**	**55.2 ± 22.9**	**p=0.94**
Age of onset ± SD	**62 ± 14.8**	**62 ± 18**	**p=0.15**
Duration of breathlessness (median years / IQR)	**8 (10.5)**	**9.5(10)**	**p=0.85**
Resting SpO_2% (median / IQR)	**96 (3)**	**94 (3)**	**p=0.003**
Exacerbation frequency (median/IQR)	**0.5 (3)**	**0 (2)**	**p=0.057**

Supplementary Table S5. Logistic regression coefficients for comparison of brain activity in response to fearful or happy faces between high and low symptom load groups. (p values family wise error corrected p<0.05).

	Coefficient	Corrected P-value
Fear	2.01	p=1.0
Happy	-0.49	p=1.0
Fear > Happy	-2.11	p=1.0
Happy > Fear	-0.75	p=1.0

Supplementary Table S6. Null hypothesis testing. Results compare brain activity in response to fearful and happy faces to the null hypothesis that there was no significant brain activity in response to these stimuli (p values family wise error corrected p<0.05).

	Mean	Corrected p-value
Fear	0.11	p<0.001
Happy	0.10	p<0.001

No significant differences in brain activity were observed in response to any of the four faces contrasts between the two groups. However, both fearful and happy faces were found to significantly activate the amygdala (p<0.001). This suggests that although there was no difference in reactivity to emotional faces between the two groups or between the two conditions (happy versus fearful), the task itself was producing a change in brain activity.

Further characterization of the fMRI results can be found in supplementary Figure S7, where the response to breathlessness-related words compared to non-words in all participants is shown.

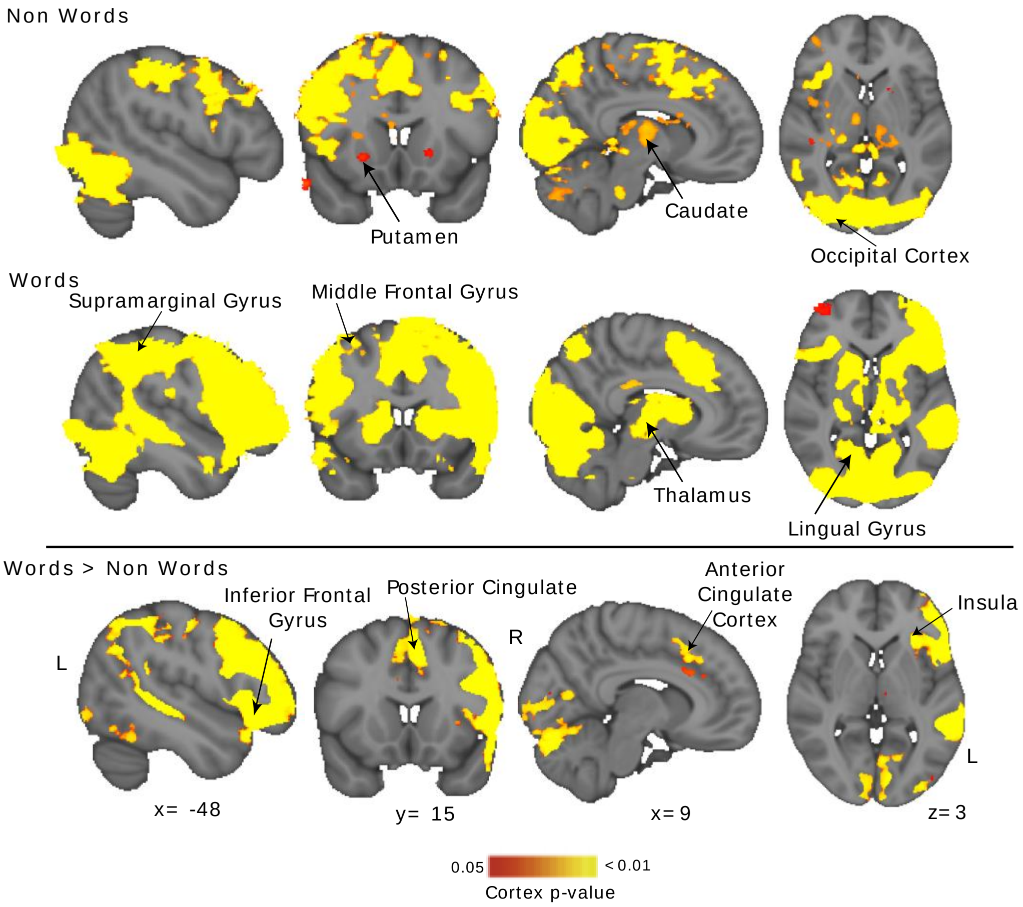

Supplementary Figure S7. Blood Oxygen Level Dependent (BOLD) activity in response to breathlessness related words, non-words and the contrast of breathlessness-related words > non-words across all participants. Results are displayed with a non-parametric TFCE p < 0.05.

Additional exploration of brain imaging data

To examine the relationship between scores across the four factors and brain activity we entered the scores for each participant as additional covariates into the group level analysis in which mean voxelwise difference in activity were calculated for the breathlessness-related words > non-words and non-words > breathlessness-related words. An F-test was performed across the four factors to account for correlations between the regressors. Demeaned age and sex values were modeled as regressors

of no interest. Significance testing was performed using FSL's Randomize tool, which carries out rigorous permutation testing, with threshold free cluster enhancement (TFCE) (a non-parametric test) at family wise error corrected $p<0.05$. The results of this analysis showed no significant brain activity associated with any of the individual factors. It is important to note that given the significant correlations between factors, such an analysis would require very careful interpretation as the factors are not independent.

In an additional visualisation of the data, peak voxel coordinates were extract for each of the 12 brain regions active in response to breathlessness-related words > non-words across both groups. These seed coordinates were expanded to form a 5mm sphere from which percentage BOLD activity, zscore and standard deviations were extracted for each region from each group (Supplementary Table S7).

Supplementary Table S7. Contains coordinates for peak voxels in response to breathlessness-related word cues across both groups for regions shown as active in Figure 5, panel A. Mean brain activity across the low and high symptom load groups is recorded as mean zstat and percentage BOLD along with standard deviation (SD).

Region	Peak voxel			Low symptom load (mean z-score ± SD)	High symptom load (mean z-score ± SD)	Low symptom load (%BOLD ± SD)	High symptom load (%BOLD ± SD)
	x	y	z				
Inferior frontal gyrus	56	16	1	0.27 ± 0.84	-0.11 ± 0.73	1.40 ± 4.39	-0.64 ± 4.07
Middle frontal gyrus	-50	8	43	0.82 ± 0.90	0.39 ± 0.87	3.78 ± 4.19	1.76 ± 4.68
Paracingulate gyrus	0	47	29	0.25 ± 0.67	-0.22 ± 0.66	1.46 ± 3.74	-0.73 ± 4.09
Anterior insula	-41	18	-7	0.59 ± 0.84	-0.03 ± 0.94	3.09 ± 4.56	-0.72 ± 6.37
Temporal pole	-44	18	-9	0.86 ± 1.01	0.05 ± 0.84	5.16 ± 6.13	-0.43 ± 6.99
Precuneus	-3	-44	55	-0.09 ± 0.72	-0.30 ± 1.00	-0.27 ± 2.96	-2.16 ± 8.17
Posterior cingulate cortex	-2	-30	46	0.05 ± 0.72	-0.27 ± 0.81	0.29 ± 2.91	-1.56 ± 5.66
Anterior cingulate cortex	3	42	29	0.22 ± 0.71	-0.14 ± 0.75	1.34 ± 3.88	-0.25 ± 4.68
Supramarginal gyrus	-63	-25	23	-0.08 ± 0.82	-0.42 ± 0.84	-0.15 ± 3.61	-2.23 ± 3.89
Thalamus	5	-13	2	0.22 ± 0.74	-0.17 ± 0.73	1.82 ± 5.68	-1.12 ± 5.01
Superior frontal gyrus	2	52	29	0.22 ± 0.85	-0.27 ± 0.88	1.72 ± 5.22	-1.07 ± 5.61
Frontal gyrus	16	56	38	0.18 ± 0.76	-0.04 ± 0.86	1.14 ± 4.32	-0.38 ± 5.22

References

1. Yorke, J., et al., *Quantification of dyspnoea using descriptors: development and initial testing of the Dyspnoea-12.* 2010. **65**(1): p. 21-26.
2. Radloff, L.S., *The CES-D Scale:A Self-Report Depression Scale for Research in the General Population.* 1977. **1**(3): p. 385-401.
3. Spielberger, C.D., *State-Trait Anxiety Inventory*, in *The Corsini Encyclopedia of Psychology*. 2010.
4. Krupp, L.B., et al., *The fatigue severity scale: Application to patients with multiple sclerosis and systemic lupus erythematosus.* Archives of Neurology, 1989. **46**(10): p. 1121-1123.
5. Jones, P.W., et al., *A self-complete measure of health status for chronic airflow limitation. The St. George's Respiratory Questionnaire.* Am Rev Respir Dis, 1992. **145**(6): p. 1321-7.
6. Bestall, J.C., et al., *Usefulness of the Medical Research Council (MRC) dyspnoea scale as a measure of disability in patients with chronic obstructive pulmonary disease.* Thorax, 1999. **54**(7): p. 581-586.
7. Chambless, D.L., et al., *The Mobility Inventory for Agoraphobia.* Behav Res Ther, 1985. **23**(1): p. 35-44.
8. De Peuter, S., et al., *Illness-specific catastrophic thinking and overperception in asthma.* Health Psychol, 2008. **27**(1): p. 93-9.
9. Herigstad, M., et al., *Dyspnea-related cues engage the prefrontal cortex: evidence from functional brain imaging in COPD.* Chest, 2015. **148**(4): p. 953-961.
10. McCracken, L.M., K.E. Vowles, and C. Eccleston, *Acceptance-based treatment for persons with complex, long standing chronic pain: a preliminary analysis of treatment outcome in comparison to a waiting phase.* Behav Res Ther, 2005. **43**(10): p. 1335-46.
11. Levy, M.L., et al., *Diagnostic spirometry in primary care: Proposed standards for general practice compliant with American Thoracic Society and European Respiratory Society recommendations: a General Practice Airways Group (GPIAG)1 document, in association with the Association for Respiratory Technology & Physiology (ARTP)2 and Education for Health3 1 www.gpiag.org 2 www.artp.org 3 www.educationforhealth.org.uk.* Prim Care Respir J, 2009. **18**(3): p. 130-47.
12. Bradley, J., et al., *Validity of a modified shuttle test in adult cystic fibrosis.* Thorax, 1999. **54**(5): p. 437-9.
13. Mahler, D.A., et al., *Comparison of clinical dyspnea ratings and psychophysical measurements of respiratory sensation in obstructive airway disease.* Am Rev Respir Dis, 1987. **135**(6): p. 1229-33.
14. Herigstad, M., et al., *Development of a dyspnoea word cue set for studies of emotional processing in COPD.* Respiratory physiology & neurobiology, 2016. **223**: p. 37-42.
15. Ekman, P.F.W., *Pictures of Facial Affect.* Consult. Psychol. Press, 1975.
16. Young, A.W., et al., *Facial expression megamix: Tests of dimensional and category accounts of emotion recognition.* Cognition, 1997. **63**(3): p. 271-313.
17. Godlewska, B.R., et al., *Short-term SSRI treatment normalises amygdala hyperactivity in depressed patients.* Psychological Medicine, 2012. **42**(12): p. 2609-2617.

18. Rawlings, N.B., et al., *A single dose of mirtazapine modulates neural responses to emotional faces in healthy people.* Psychopharmacology, 2010. **212**(4): p. 625-634.
19. Oliveira, L., et al., *What does brain response to neutral faces tell us about major depression? evidence from machine learning and fMRI.* PloS one, 2013. **8**(4): p. e60121-e60121.
20. Fraley, C. and A.E. Raftery, *Model-Based Clustering, Discriminant Analysis, and Density Estimation.* Journal of the American Statistical Association, 2002. **97**(458): p. 611-631.
21. Saraçli, S., N. Doğan, and İ. Doğan, *Comparison of hierarchical cluster analysis methods by cophenetic correlation.* Journal of Inequalities and Applications, 2013. **2013**(1): p. 203.
22. Ward, J.H., *Hierarchical Grouping to Optimize an Objective Function.* Journal of the American Statistical Association, 1963. **58**(301): p. 236-244.
23. Costello, A.B. and J. Osborne, *Best practices in exploratory factor analysis: Four recommendations for getting the most from your analysis.* Practical assessment, research, and evaluation, 2005. **10**(1): p. 7.
24. Zygmont, C. and M. Smith, *Robust factor analysis in the presence of normality violations, missing data, and outliers: Empirical questions and possible solutions.* The Quantitative Methods for Psychology, 2014. **10**: p. 40-55.
25. Schreiber, J.B., et al., *Reporting Structural Equation Modeling and Confirmatory Factor Analysis Results: A Review.* The Journal of Educational Research, 2006. **99**(6): p. 323-338.
26. Rosseel, Y., *lavaan: An R Package for Structural Equation Modeling.* 2012, 2012. **48**(2): p. 36.
27. Jenkinson, M., et al., *Improved optimization for the robust and accurate linear registration and motion correction of brain images.* Neuroimage, 2002. **17**(2): p. 825-41.
28. Smith, S.M., *Fast robust automated brain extraction.* Hum Brain Mapp, 2002. **17**(3): p. 143-55.
29. Holland, D., J.M. Kuperman, and A.M. Dale, *Efficient correction of inhomogeneous static magnetic field-induced distortion in Echo Planar Imaging.* Neuroimage, 2010. **50**(1): p. 175-83.
30. Jenkinson, M., *Fast, automated, N-dimensional phase-unwrapping algorithm.* Magn Reson Med, 2003. **49**(1): p. 193-7.
31. Greve, D.N. and B. Fischl, *Accurate and robust brain image alignment using boundary-based registration.* Neuroimage, 2009. **48**(1): p. 63-72.
32. Griffanti, L., et al., *ICA-based artefact removal and accelerated fMRI acquisition for improved resting state network imaging.* Neuroimage, 2014. **95**: p. 232-47.
33. Salimi-Khorshidi, G., et al., *Automatic denoising of functional MRI data: combining independent component analysis and hierarchical fusion of classifiers.* Neuroimage, 2014. **90**: p. 449-68.
34. Filippini, N., et al., *Study protocol: the Whitehall II imaging sub-study.* BMC Psychiatry, 2014. **14**(1): p. 159.
35. Harvey, A.K., et al., *Brainstem functional magnetic resonance imaging: disentangling signal from physiological noise.* J Magn Reson Imaging, 2008. **28**(6): p. 1337-44.

36. Brooks, J.C., et al., *Physiological noise modelling for spinal functional magnetic resonance imaging studies.* Neuroimage, 2008. **39**(2): p. 680-92.
37. Faull, O.K., et al., *Conditioned respiratory threat in the subdivisions of the human periaqueductal gray.* Elife, 2016. **5**.
38. Hayen, A., et al., *Opioid suppression of conditioned anticipatory brain responses to breathlessness.* Neuroimage, 2017. **150**: p. 383-394.
39. Andersson, J., *Non-Linear registration, aka spatial normalisation.* FMRIB technical report, 2010. **TR07JA2**.
