# Supplementary material for: Breathlessness in COPD: linking symptom clusters with brain activity
Source: Eur Respir J. 2021 Nov 18;58(5):2004099. doi: 10.1183/13993003.04099-2020 (PMC8607925; doi:10.1183/13993003.04099-2020)
Supplement: Supplementary file 2 [file ERJ-04099-2020.Shareable.pdf]

EUROPEAN RESPIRATORY JOURNAL
ORIGINAL RESEARCH ARTICLE
S.L. FINNEGAN ET AL.

SHAREABLE PDF

Breathlessness in COPD: linking symptom clusters with brain activity

Sarah L. Finnegan[1], Olivia K. Harrison[1,2,3], Catherine J. Harmer[4,5], Mari Herigstad[6], Najib M. Rahman[7,8], Andrea Reinecke[4] and Kyle T.S. Pattinson[1]

[1]Wellcome Centre for Integrative Neuroimaging and Nuffield Division of Anaesthetics, Nuffield Dept of Clinical Neurosciences, University of Oxford, Oxford, UK. [2]Translational Neuromodeling Unit, Institute for Biomedical Engineering, University of Zurich and ETH Zurich, Zurich, Switzerland. [3]School of Pharmacy, University of Otago, Dunedin, New Zealand. [4]Dept of Psychiatry, Medical Sciences Division, University of Oxford, Oxford, UK. [5]Oxford Health NHS Foundation Trust, Warneford Hospital, Oxford, UK. [6]Dept of Biosciences and Chemistry, Sheffield Hallam University, Sheffield, UK. [7]Nuffield Dept of Medicine, University of Oxford, Oxford, UK. [8]NIHR Oxford Biomedical Research Centre, Oxford, UK.

Corresponding author: Sarah L. Finnegan (sarah.finnegan@ndcn.ox.ac.uk)

Check for updates

Shareable abstract (@ERSpublications)
Towards individualised treatments for chronic breathlessness with functional neuroimaging: revealing the factors underlying the breathlessness experience in COPD https://bit.ly/3a8fXPt

Cite this article as: Finnegan SL, Harrison OK, Harmer CJ, *et al*. Breathlessness in COPD: linking symptom clusters with brain activity. *Eur Respir J* 2021; 58: 2004099 [DOI: 10.1183/13993003.04099-2020].

This single-page version can be shared freely online.

Copyright ©The authors 2021.

This version is distributed under the terms of the Creative Commons Attribution Licence 4.0.

This article has supplementary material available from erj.ersjournals.com

Received: 28 April 2020
Accepted: 4 April 2021

Abstract

Background Current models of breathlessness often fail to explain disparities between patients' experiences of breathlessness and objective measures of lung function. While a mechanistic understanding of this discordance has thus far remained elusive, factors such as mood, attention and expectation have all been implicated as important modulators of breathlessness. Therefore, we have developed a model to better understand the relationships between these factors using unsupervised machine learning techniques. Subsequently we examined how expectation-related brain activity differed between these symptom-defined clusters of participants.

Methods A cohort of 91 participants with mild-to-moderate chronic obstructive pulmonary disease (COPD) underwent functional brain imaging, self-report questionnaires and clinical measures of respiratory function. Unsupervised machine learning techniques of exploratory factor analysis and hierarchical cluster modelling were used to model brain–behaviour–breathlessness links.

Results We successfully stratified participants across four key factors corresponding to mood, symptom burden and two capability measures. Two key groups resulted from this stratification, corresponding to high and low symptom burden. Compared with the high symptom burden group, the low symptom burden group demonstrated significantly greater brain activity within the anterior insula, a key region thought to be involved in monitoring internal bodily sensations (interoception).

Conclusions This is the largest functional neuroimaging study of COPD to date, and is the first to provide a clear model linking brain, behaviour and breathlessness expectation. Furthermore, it was possible to stratify participants into groups, which then revealed differences in brain activity patterns. Together, these findings highlight the value of multimodal models of breathlessness in identifying behavioural phenotypes and for advancing understanding of differences in breathlessness burden.
